# Microsatellite Instability in Chicken Lymphoma Induced by Gallid Herpesvirus 2

**DOI:** 10.1371/journal.pone.0068058

**Published:** 2013-07-02

**Authors:** Da-Wei Yao, Jia-Rong Xu, Zhen-Lei Zhou, Shang-Tong Li, De-Ji Yang

**Affiliations:** College of Veterinary Medicine, Nanjing Agricultural University, Nanjing, Jiangsu, China; Hannover Medical School, Germany

## Abstract

Microsatellite instability (MSI) has been found in a range of human tumors, and little is known of the links between MSI and herpesvirus. In order to investigate the relationship between MSI and Gallid herpesvirus 2 (GaHV-2)-induced lymphoma, fifteen Marek’s disease (MD) lymphomas were analyzed through using 46 microsatellite markers, which were amplified by PCR from DNA specimens of lymphoma and normal muscular tissues from the same chicken. PCR products were evaluated by denaturing polyacrylamide gel electrophoresis for MSI analysis. MSI was proved in all lymphomas, at least in one locus. Thirty of the 46 microsatellite markers had microsatellite alterations. These results suggested that GaHV-2-induced lymphoma in chickens is related to MSI, and this is the first report to demonstrate that MSI is associated with the GaHV-2 induced lymphoma in chicken.

## Introduction

Herpesviruses are important pathogens associated with a wide range of disease in human beings and animals,and some of them are associated with cancer in their natural hosts. Epstein-Barr virus (EBV) is the etiological agent of nasopharyngeal carcinoma (NPC), African Burkitt’s lymphoma, posttransplant lymphomas (PTLD), Hodgkin’s disease, and some gastric cancers [1], [2]. Kaposi’s sarcoma-associated herpesvirus (KSHV) is linked to Kaposi sarcoma (KS) and two lymphoproliferative diseases, *i.e.* primary effusion lymphoma and multicentric Castleman’s disease [1], [2]. Marek’s disease (MD), which is caused by Gallid herpesvirus 2 (GaHV-2), is characterized by visceral T-cell lymphomas, paralysis, blindness, and neurological dysfunction in chicken [3]. Apart from an economically important disease affecting poultry health and welfare, MD is a good model for studying the pathogenesis and immune control of herpesvirus-induced oncogenicity [4], [5]. Several MDV-encoded genes related to oncogenesis have been identified, such as major oncogene *meq* (Marek’s EcoR I-Q) and viral telomerase RNA [6]. MEQ is considered to be the major viral oncoprotein of MDV and can induce transcriptional activation or repression depending on its dimerization partner and DNA binding specificity. The host response to MDV infection has been analyzed by proteomic and transcriptomic approaches. Differentially expressed proteins were mainly associated with tumor biology, protein folding, signal transduction, immunology, cell proliferation and apoptosis [7]. Studying host responses to pathogens on the gene or protein level has contributed to our understanding of various host–pathogen interactions.

Microsatellite instability (MSI) is defined as a change of any length caused by either the insertion or deletion of repeating units, in a microsatellite within a tumor when compared to normal tissue [8]. It has been variously reported in a range of human tumor types, including lung [9], bladder [10], ovarian [11], colorectum [8], and breast [12] and it is the hallmark of mismatch repair (MMR) system deficiency. Loss of MMR may contribute to tumorigenesis by elevating both the rate of mutations and mitotic recombination.

The information available regarding the relationships between herpesvirus and MSI in carcinogenesis is controversial. Chang et al found that EBV-positive cases in gastric carcinomas showed no MSI positivity [13], [14]. However, Wu et al and Leung et al found MSI positivity in both EBV-negative and EBV-positive gastric carcinomas [15], [16]. In order to evaluate the involvement of GaHV-2 and MSI in Marek’s disease lymphoma, forty-six microsatellite markers, which showed a high frequency of MSI in primary chicken embryo fibroblasts infected with GaHV-2 in our previous research, were selected from 304 markers to evaluate the frequency of MSI.

## Materials and Methods

### Experimental Animals and Infective Virus Strain

Specific pathogen free (SPF) White Leghorn Chickens (Merial Vital Laboratory Animal Technology Co., Ltd, China) were kept at the animal isolation facility at Nannong Hi-Tech Co., Ltd (Nanjing, China). A virulent strain of MDV (GaHV-2) J-1 at passage 32, kindly provided by Merial Animal Health Co., Ltd (Shanghai, China), was used to infect the chickens.

### Experimental Design

After 21 days post-hatch, forty SPF White Leghorn chickens were intraabdominally inoculated with 1,000 plaque-forming units of MDV J-1 and housed in the isolation facility in separate rooms. All birds were evaluated daily for symptoms of MD, and were examined for gross MD lesions. Chickens which present ataxia or paralysis and moribund state were euthanized by CO_2_. The experiment was terminated 58 days after infection. All animal experiments were carried out in dedicated negative pressure rooms and conducted strictly in accordance with the laboratory animal guidelines. The protocol was approved by Laboratory Animal Management Committee of Jiangsu Province.

### Histopathological Examination

Tissues samples were removed and collected from chicken euthanized during the experiment period and termination, including the liver, spleen, kidney, heart, lung, peripheral nerves, skin, gonads, thymus and bursa of fabricius. Different tissue samples were fixed in 10% formalin and embedded in paraffin wax via a routine process. All sections were stained with hematoxylin and eosin (H&E) and histopathologically examined through using an optical microscope.

### Detection of Microsatellite Instability

Microsatellite instability was detected through using gel-based nonradioactive methods described by Shang et al. [17].

#### Sample collection and genomic DNA extraction

Tumor specimens that were verified visually and histopathologically were collected for MSI analysis. The genomic DNA of tumors and normal muscular tissue samples from the same chicken were extracted with TIANamp Genomic DNA kit (TIANGEN, China), according to the manufacturer’s instructions. Their concentrations were determined by using a BioPhotometer plus (Eppendorf, Germany).

#### Polymerase chain reaction for amplification of microsatellite markers

Forty-six microsatellite markers (Table 1), which showed a high frequency of MSI in primary chicken embryo fibroblasts infected with GaHV-2 in our previous research [18], were selected from 304 markers to evaluate the frequency of MSI. PCR reaction mixtures contained the following components: 1 µl genomic DNA template (50 ng/µl), 1 µl of Taq DNA polymerase (5 U/µl), 5 µl of 10× PCR buffer, 1 µl of each primer (10 µmol/L), 4 µl of dNTPs (2.5 mmol/L), and 37 µl water. Touchdown PCR amplification was performed in a PTC-200 (Bio-Rad, USA). The initial touchdown cycle comprised denaturation at 96°C for 30 s, annealing at 65°C for 30 s, and extension at 72°C for 30 s. During the touchdown phase, the annealing temperature was decreased at the rate of 1°C for every cycle of the amplification reaction. After 10 touchdown cycles, 25 standard PCR cycles were performed under the following conditions: 96°C for 30 s, annealing at 55°C for 30 s, extension at 72°C for 30 s, and a final extension at 72°C for an additional 5 mins. The PCR reaction was terminated by adding 20 µl of gel loading dye (98% formamide, 10 mmol/L EDTA, and 0.05% bromophenol blue).

**Table 1:** Microsatellite markers used in this study.

*Marker name*	*Chromosome*	*Primer (5′-3′)*	*Size (bp)*	*Repeat array*
MCW0248	1	GTTGTTCAAAAGAAGATGCATG	220–225	(CA) 9
		TTGCATTAACTGGGCACTTTC		
ABR0352	1	AAACCTCGGCCACGTCCATC	334	(CA) 9
		GGAATTAACCACCGCCACCAG		
ABR0329	1	TTCCCAGAGTCACTCATCTC	314	(TA) 17
		TTCATGGTGTATTTCTCCTG		
ABR0522	1	GAATTTAGGAGGCTTTGTCC	193	(CA) 9
		CTTTTGTGCATTTGTGGGTT		
MCW0145	1	ACTTTATTCTCCAAATTTGGCT	212	(CAA) 6 (CA) 20
		AAACACAATGGCAACGGAAAC		
LEI0146	1	TCAAGCCACCAAAGTGCTTGG	276	(AC)22
		GATCACTCTGCTCATAGCAGT		
ABR0204	1	TAAATAAAGGTGTTGGCAGTT	280	(CA) 20
		CAGATTGTTAAAATAGTTGGGTT		
ABR0007	1	ACACCATCCATTATGAACAC	115	(CA) 11
		AACAATATGACCATTAACTGC		
MCW0115	1	ATACCAACATCTGCCTGTGAC	252	(CA) 18
		GCAGTGTGTCTGACTAGCTCT		
ABR0004	2	CAATAGCAATGCCAAATGAAAC	99	(CA) 8
		GCAAGTTGATGTCCGTGGTG		
ABR0008	2	TAAGTGATGCGACGGGAAAG	265	(CA) 14
		CGCTGAGATGGAACAAAGGAG		
ABR0107	2	CCGTTACTGACTTCTGCTTT	250	(CA) 10
		TTTGTATTGGCTCCCTCATC		
ABR0189	2	TTAACATTAAGAGCGCATCT	109	(CA) 9
		ATTTGAACTTCCAAAACACT		
ABR0153	2	GACAGACCCACTACTGCTGA	263	(CA) 8
		TACCCTAACTGTTCCCACAA		
ABR0659	2	AAGCAACAACGTGCCTACAA	284	(CA) 12
		GACTATCAAGATATTCACCAAA		
ABR0382	4	CTTGTTGTGGAACCCTTAGT	226	(CA) 9
		TGAGAATCTGGCCTGATATT		
ABR0622	4	GAGCTCCACTCTACCCCATG	231	(CA) 15
		TCTTGCCAACTCCAGTCCTA		
ADL0266	4	AATGCATTGCAGGATGTATG	113–136	(CA) 6
		GTGGCATTCAGGCAGAGCAG		
ABR0392	5	CGAAATACAGCCCTAAGAAC	138	(CA) 9
		AATCCCTGTGAAGGAATCTA		
ABR0399	5	AGCCTAAGCATTTGAGAACA	291	(CA) 18
		GACAAGTCAAACCACGAAAC		
ABR0262	5	ATAGCAGCAGGCTCAAATGG	279	(CA) 9
		TTAATGGATGTAAAGGCAAA		
ABR0048	6	ATTCTGGGGACATCTGTGAACAC	91	(CA) 11
		CTGATACCTTTCAGCTGGTTGTG		
MCW0134	9	GGAGACTTCATTGTGTAGCAC	284	(CA) 24
		ACCAAAAGACTGGAGGTCAAC		
ABR0526	9	TCAATTCAGTACGTCCCACA	181	(CA) 10
		GCAGGAGCTGCCTATTACAT		
ABR0495	10	TTGTACTGGGTAGCATTTGA	249	(CA) 15
		ACTCTTTGGCCTACTTTTCC		
MCW0067	10	GCACTACTGTGTGCTGCAGTTT	178–184	(CA) 11
		GAGATGTAGTGCCACATTCCGAC		
ABR0325	10	CATTCTGTTTTCATTTCTGAT	156	(CA) 18
		ACGTGCTGCACTAATTTTAC		
ABR0389	11	AAAGTGCCAGACTCAACAAG	231	(CA) 12
		TTCCCTCTATCAGCATCCATCC		
ADL0308	11	CCTCTGAATGTCTGAATGAC	164–165	(CA) 13
		GGATGACTCCTTGGCAACAC		
ABR0052	11	CTGACAGAGCCTCAAAGGATAAT	209	(CA) 9
		TCGGCATGTGCTGACAAACA		
ABR0059	12	ACAAACAAGCAAGGGCCAACTAA	192	(CA) 9
		GCTGAGGAAGCAGCGGGTAA		
ABR0086	12	TGAACAGTTGTGCTGTCCAAGTT	223	(CA) 9
		CCCCGAAATGCTAAAAGAATGTC		
ABR0033	12	AAGAGGGGAGGAGGAAGCAGG	202	(CA) 10
		GCCTTTGCACGCATACACCAG		
LEI0099	12	GATCTGGCAGAACAGAAACAG	131	(CA) 12
		ATATTTCACACCTGACCTGCG		
ABR0634	12	TACTGAATAAAAGGAGGAAC	306	(CA) 21
		AATAGCCAAATAGGTACAGC		
ADL0147	13	CTGGTGAATGAGAAGCGATG	211–220	(CA) 8
		GCTGCGGCAATAAACTCCCT		
ABR0365	14	ACAGGTACAACTTTATGCAAT	222	(CA) 23
		AGCTAGGAAAAGAGGAAATA		
ABR0517	14	GCAGGATGCCTGGCAGAGGT	243	(CA) 9
		GGCCACCATCAGCCCCACGT		
ABR0387	17	AATGTGAGGTGCTGAATGGA	288	(CA) 14
		CTGTTGCCTGCCACAAATGG		
ADL0199	17	ACAAAGCCAGAGGAAACATC	154–174	(CA) 14
		GACGAAAGCAAGAGCAAAGC		
ABR0650	18	CTGAAAGAAGCAGTAAAATG	318	(CA) 11
		ATGGAAATGTGCCTTGGAGA		
ABR0133	19	CCTGGTAATGTCTGCGTTTG	199	(CA) 9
		GGAGCCGTTTCTGTATGTTT		
ABR0180	19	ATGGAATTTTACCACTGCTA	145	(CA) 11
		AAATGAATCAGACAGGGAAT		
ABR0026	20	CCGTCATCCTTCATCCGCCACA	193	(CA) 10
		AGCGCTGGGTGCTCCGGGTGT		
ABR0123	20	ACTCCAACGCCTACCAGTCA	179	(CA) 7
		CCATAACACCAAGCCATCAA		
ABR0617	26	CCAAGAACTCACATCAACGAGCAA	172	(CA) 13
		TGGAAGACTGGCAGGGAAGC		

#### Microsatellite instability analysis

PCR products were denatured at 95°C for 5 mins with gel loading dye, and put immediately on ice for 5 mins prior to loading. About 3 µl of the PCR products were loaded onto a 12% denaturing polyacrylamide gel. The gels were run on the DCode universal mutation detection system (Bio-Rad, USA) in 1×TBE at 45°C at 180V. After electrophoresis, the gels were stained with AgNO_3_ according to the method of Sanguinetti et al [19].

## Results

### Symptoms after Inoculation

Clinical signs of infection by GaHV-2 appeared among the treated chickens, including loss of appetite, depression, pallor, and paralysis of the wings, legs and necks. Death occurred in the second week after post-infection, and reached a peak during the 4th and 5th week. Morbidity was 75%, and mortality was 73.3% when the experiment was terminated. Pathological examination of the chickens tissues revealed that fifteen chickens (15/40, 37.5%) had significantly widespread, diffuse lymphomas involving the liver (n = 9), gonads (n = 12), spleen (n = 9), kidneys (n = 15), proventriculus (n = 6) and heart (n = 9). Tumors were found in twelve chickens after death or euthanasia and in three chickens after euthanasia at the predetermined time point. Fifteen kidney tumors were used to microsatellite instability analysis.

### Microsatellite Instability Screening

**Figure 1:**
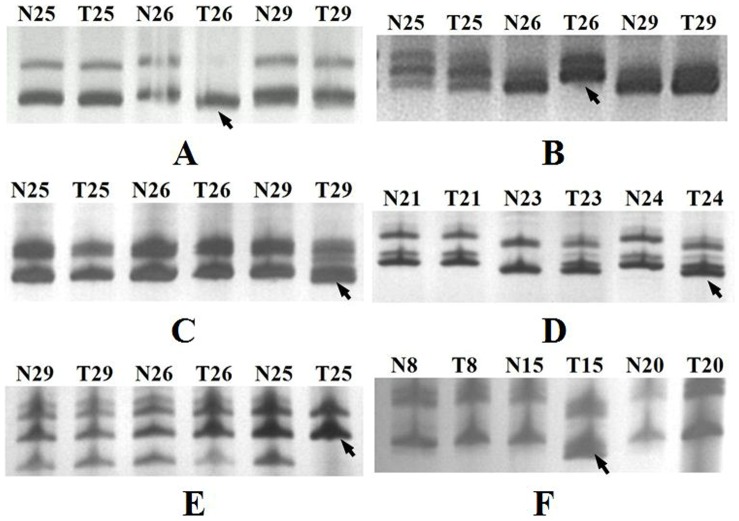
Denaturing polyacrylamide gel showing MSI in fifteen kidney tumors induced by GaHV-2 using six microsatellite markers. (A) ABR0007. (B) ABR0086. (C) ABR0352. (D) ABR0387. (E) ABR0399. (F) MCW0145. Line N, normal. Line T, tumors.

Alterations of microsatellite markers in the GaHV-2-infected samples were identified as differences in the electrophoretic migration or loss of major band(s) in comparison with normal muscular tissue samples DNA (Fig. 1). MSI was detected at one or more loci in all the tumors’ analysis (15/15, Table 2). Chicken no. 24 had nine loci (9/30, 30%) displaying MSI. Only one MSI locus was found in chicken nos. 5, 21, and 39.

**Table 2:** Number of MSI loci in different chickens.

*Chicken*	*Microsatellite markers*	*Chromosome*	*Number of MSI loci*
1	ABR0352, ABR0522, ABR0622, ABR0086, ABR0365,	1, 1, 4, 12, 14	5/30
2	ABR008, ABR0399	1, 5	2/30
5	ABR0352	1	1/30
8	ABR0107, ABR0399, ABR0133	2, 5, 19	3/30
15	MCW0145, ABR0526	1, 9	2/30
20	ABR0008, ABR0262, ABR0389, ABR0086 ABR0517	2, 5,11, 12,	5/30
21	ABR0086	2	1/30
23	ABR0352, ABR0189, ABR0659, ADL0147, ABR0517	1, 2, 2, 13, 14	5/30
24	ABR0007, ABR0352, ABR0522, MCW0145, LEI0146, ABR0052, ABR0033,ABR0634, ABR0387	1, 1, 1, 1, 1, 11, 12,12,17	9/30
25	MCW0145, ABR0399, ABR0389, ABR0033, LEI0099	1, 5, 11, 12, 12	5/30
26	MCW0145, ABR0659, ADL0266, ABR0086, ABR0634	1, 2, 4, 12, 12	5/30
29	ABR0007, ABR0204, ABR0352, ABR0617	1, 1, 1,12	4/30
37	ABR0107, ABR0387, ABR0026, ABR0123, ABR0617	2, 17, 20, 20, 26	5/30
38	ABR0204, MCW0067	1, 10	2/30
39	ABR0387	17	1/30

The frequency of MSI for each marker is shown in Table 3. Microsatellite alterations exist in 30 markers among 46 microsatellite markers. In the tumor samples, the marker that showed the highest frequency of instability (5/15, 33%) was ABR0352. Microsatellite instability was displayed at ABR0086 and MCW0145 in four tumors (27%), and at ABR0007, ABR0387, and ABR0399 in three tumors (20%).

**Table 3:** Table **3.** Frequency of MSI for each microsatellite marker.

*Microsatellite markers*	*No. of Chickens*	*Frequency*	*Microsatellite markers*	*No. of Chickens*	*Frequency*
ABR0007	2, 24, 29	3/15	ABR0389	20, 25	2/15
ABR0008	20	1/15	ABR0399	2, 8, 25	3/15
ABR0026	37	1/15	ABR0517	20, 23	2/15
ABR0033	24, 25	2/15	ABR0522	1, 24	2/15
ABR0052	24	1/15	ABR0526	15	1/15
ABR0086	1, 20, 21, 26	4/15	ABR0617	29, 37	2/15
ABR0107	37, 8	2/15	ABR0622	1	1/15
ABR0123	37	1/15	ABR0634	24, 26	2/15
ABR0133	8	1/15	ABR0659	23, 26	2/15
ABR0189	23	1/15	ADL0147	23	1/15
ABR0204	29, 38	2/15	ADL0266	26	1/15
ABR0262	20	1/15	MCW0067	38	1/15
ABR0352	1, 5, 23, 24, 29	5/15	MCW0145	15, 24, 25, 26	4/15
ABR0365	1	1/15	LEI0099	25	1/15
ABR0387	24, 37, 39	3/15	LEI0146	24	1/15

## Discussion

Since the initial description of MSI in HNPCC in 1993, MSI has been identified in a wide variety of human cancers, both familial and sporadic [20]. Microsatellite instability (MSI) is a form of genomic instability. Higher MSI frequency is a prominent genetic feature in many tumors. However, there is no study of MSI in Marek’s Disease lymphoma induced by GaHV-2. In this study, we collected MD lymphoma tissue specimens, and evaluated MSI frequencies through using 46 microsatellite markers. All lymphoma showed microsatellite instability in at least one locus. These results indicated that MSI was present in Marek’s disease virus-induced lymphoma. Among three serotypes, only serotype 1 MDV (GaHV-2) causes lymphoma formation in chickens, the other two serotypes (GaHV-3 and HVT) are non-pathogenic. MD vaccine strain CVI988 or HVT is effective in the prevention of tumor development but not infection. The MDV oncoprotein Meq differs between oncogenic and vaccine strains [21]. It remains to be seen whether MSI frequencies is different in all kinds of MDV strains.

MSI has been thought to be closely related to mutation of proteins involved in the MMR system, which normally maintains a low rate of spontaneous mutations and corrects replication errors. Many studies noted the phenomenon of mutation of mismatch repair genes in the development and progression of human tumors [22], [23]. Lu et al had found that hypothetical protein (mismatch repair ATPase, MutS family) persistently up-regulated in the bursa of fabricius of chickens infected with the highly virulent strain [24]. Interaction of MDV-encoded proteins and host cell pathways will mediate cell proliferation and apoptosis. The transcriptional regulator MEQ is considered to be the major viral oncoprotein of GaHV-2. The MEQ interacts directly with p53 and inhibits p53-mediated transcriptional activity [25]. Both the MMR system and the p53 pathway are critical in the maintenance of genomic integrity [26], [27].

A panel of five microsatellites has been validated and recommended as a reference panel for future research in the field at a National Cancer Institute Workshop meeting [8]. Tumors with instability at two or more of these markers were defined as being MSI-H (high-frequency MSI), whereas those with instability at one, or showing no instability, were defined as MSI-L (low-frequency MSI) and MSS (microsatellite stable) tumors, respectively. If more than five markers are used to identify particular tumor phenotypes, then the criteria should be modified to assess the percentage of MSI rather than absolute number. The MSI-H group of tumors would be defined as having MSI in ≥30–40%, whereas the MSI-L group would exhibit MSI in <30%. The diagnosis of MSI-H in cancers is becoming increasingly relevant; MSI-H is a useful screening marker for identifying some human tumors [28]. This was the first report of MSI in Marek’s Disease lymphoma, and there is no reference to MSI-H/MSI-L markers for this tumor. Therefore, we recommend six markers (ABR0007, ABR0086, ABR0352, ABR0387, ABR0399, and MCW0145) as predictive biomarkers of Marek’s Disease lymphoma as 40% (6/15) demonstrated MSI-H and 93% (14/15) of lymphomas showed MSI in the present study, according to the human criteria detailed above.

In conclusion, this study demonstrates that the phenomenon of MSI does occur in Marek’s disease lymphoma induced by GaHV-2. Future studies will investigate the relationships among MMR, MSI and MD tumorigenesis to better understand the molecular nature of host genomic instability and virus infection.
